# Supplementary material for: Saccharomyces boulardii (CNCM I-745) improves intestinal damage in sepsis by remodeling intestinal flora structure
Source: Front Cell Infect Microbiol. 2025 Jul 31;15:1602792. doi: 10.3389/fcimb.2025.1602792 (PMC12350329; doi:10.3389/fcimb.2025.1602792)
Supplement: Supplementary file 1 [file DataSheet1.zip › summary/summary/8_Advanced_analyse/2_PICRUSt2_result/COG/Group.BW4_vs_BW0/PICRUSt2_COG_Group.BW4_vs_BW0_diff.pdf]

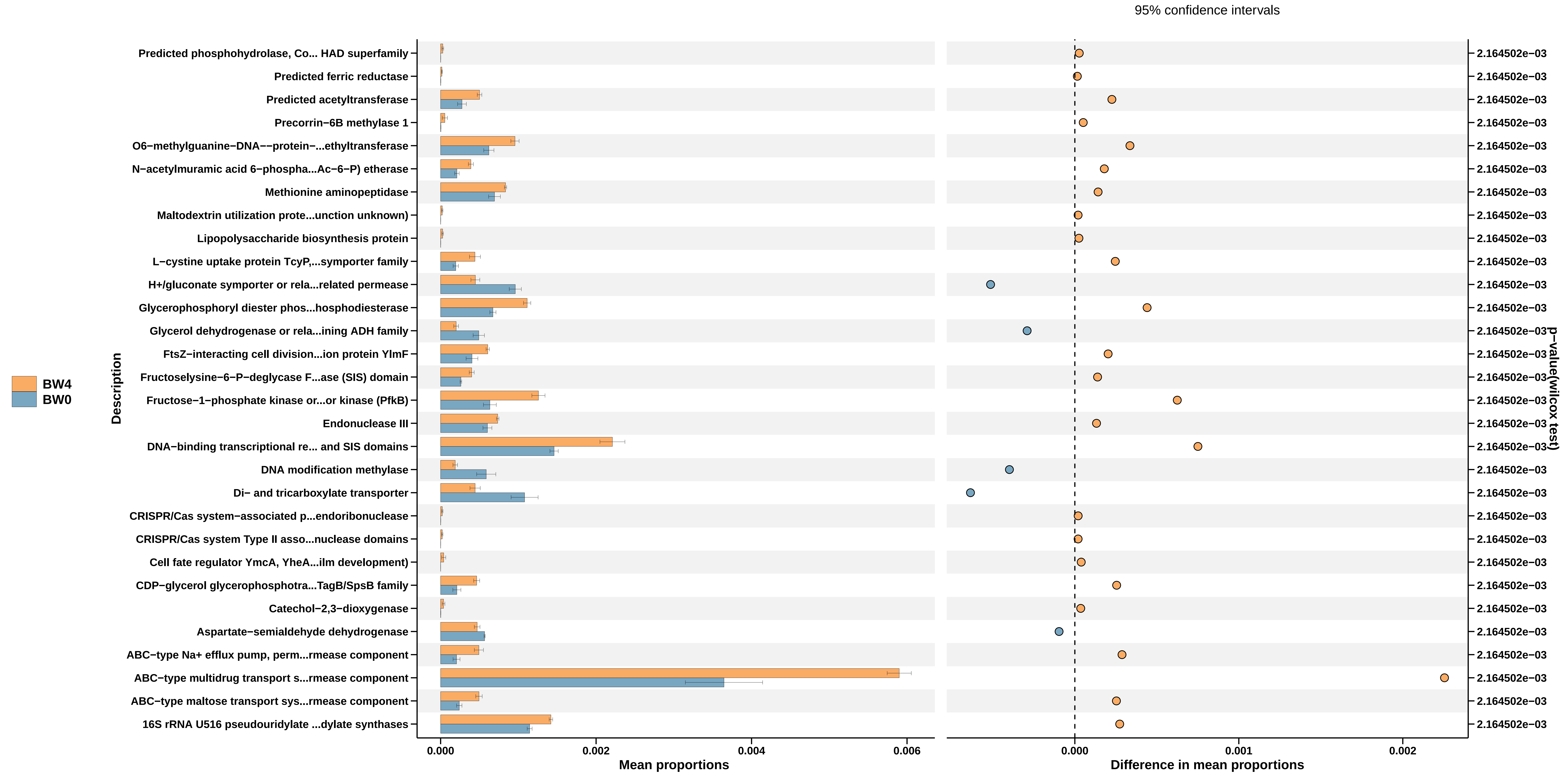
95% confidence intervals
Predicted phosphohydrolase, Co... HAD superfamily
Predicted ferric reductase
Predicted acetyltransferase
Precorrin−6B methylase 1
O6−methylguanine−DNA−−protein−...ethyltransferase
N−acetylmuramic acid 6−phospha...Ac−6−P) etherase
Methionine aminopeptidase
Maltodextrin utilization prote...unction unknown)
Lipopolysaccharide biosynthesis protein
L−cystine uptake protein TcyP,...symporter family
H+/gluconate symporter or rela...related permease
Glycerophosphoryl diester phos...hosphodiesterase
Glycerol dehydrogenase or rela...ining ADH family
FtsZ−interacting cell division...ion protein YlmF
Fructoselysine−6−P−deglycase F...ase (SIS) domain
Fructose−1−phosphate kinase or...or kinase (PfkB)
Endonuclease III
DNA−binding transcriptional re... and SIS domains
DNA modification methylase
Di− and tricarboxylate transporter
CRISPR/Cas system−associated p...endoribonuclease
CRISPR/Cas system Type II asso...nuclease domains
Cell fate regulator YmcA, YheA...ilm development)
CDP−glycerol glycerophosphotra...TagB/SpsB family
Catechol−2,3−dioxygenase
Aspartate−semialdehyde dehydrogenase
ABC−type Na+ efflux pump, perm...rmease component
ABC−type multidrug transport s...rmease component
ABC−type maltose transport sys...rmease component
16S rRNA U516 pseudouridylate ...dylate synthases
Description
BW4
BW0
0.000
0.002
0.004
0.006
Mean proportions
0.000
0.001
0.002
Difference in mean proportions
2.164502e−03
p−value(wilcox test)
